# Epidemiology and clinical profile of pathogens responsible for the hospitalization of children in Sousse area, Tunisia

**DOI:** 10.1371/journal.pone.0188325

**Published:** 2017-11-17

**Authors:** Ines Brini, Aida Guerrero, Naila Hannachi, Jihene Bouguila, Dorothea Orth-Höller, Amira Bouhlel, Lamia Boughamoura, Benjamin Hetzer, Wegene Borena, Britta Schiela, Dorothee Von Laer, Jalel Boukadida, Heribert Stoiber

**Affiliations:** 1 Laboratory of Microbiology and Immunology, Faculty of Medicine of Sousse, University of Sousse, Sousse, Tunisia; 2 Research Unit for Genomic Characterization of Infectious Agents UR12SP34, University-Hospital of Farhat Hached of Sousse, Sousse, Tunisia; 3 Faculty of Pharmacy of Monastir, University of Monastir, Monastir, Tunisia; 4 Innsbruck Medical University, Innsbruck, Austria; 5 Division of Virology, Innsbruck Medical University, Innsbruck, Austria; 6 Pediatric Service, University-Hospital of Farhat Hached of Sousse, Sousse, Tunisia; 7 Division of Hygiene and Medical Microbiology, Innsbruck Medical University, Innsbruck, Austria; Defense Threat Reduction Agency, UNITED STATES

## Abstract

This study aimed to identify a broad spectrum of respiratory pathogens from hospitalized and not-preselected children with acute respiratory tract infections in the Farhat Hached University-hospital of Sousse, Tunisia. Between September 2013 and December 2014, samples from 372 children aged between 1 month and 5 years were collected, and tested using multiplex real-time RT-PCR by a commercial assay for 21 respiratory pathogens. In addition, samples were screened for the presence of *Streptococcus pneumoniae* 16S rDNA using real-time PCR. The viral distribution and its association with clinical symptoms were statistically analyzed. Viral pathogens were detected in 342 (91.93%) of the samples of which 28.76% were single positive and 63.17% had multiple infections. The most frequent detected viruses were rhinovirus (55.64%), respiratory syncytial virus A/B (33.06%), adenovirus (25.00%), coronavirus NL63, HKU1, OC43, and 229E (21.50%), and metapneumovirus A/B (16.12%). Children in the youngest age group (1–3 months) exhibited the highest frequencies of infection. Related to their frequency of detection, RSV A/B was the most associated pathogen with patient’s demographic situation and clinical manifestations (p<0.05). Parainfluenza virus 1–4 and parechovirus were found to increase the risk of death (p<0.05). Adenovirus was statistically associated to the manifestation of gastroenteritis (p = 0.004). Rhinovirus infection increases the duration of oxygen support (p = 0.042). Coronavirus group was statistically associated with the manifestation of bronchiolitis (p = 0.009) and laryngitis (p = 0.017). *Streptococcus pneumoniae* DNA was detected in 143 (38.44%) of tested samples. However, only 53 samples had a concentration of C-reactive protein from equal to higher than 20 milligrams per liter, and 6 of them were single positive for *Streptocuccus pneumoniae*. This study confirms the high incidence of respiratory viruses in children hospitalized for acute respiratory tract infections in the Sousse area, Tunisia.

## Introduction

Acute respiratory tract infections (ARTIs) are the most common cause of childhood morbidity and mortality worldwide, accounting for about 30% of all childhood deaths in the developing world [1]. The WHO estimates that ARTIs account for 1.9 to 2.2 million childhood deaths annually, with 42% occurring in Africa [2]. The etiologic agents include viruses, bacteria, and fungi. Among the viruses responsible for ARTIs are members of the *Orthomyxoviridae*, *Coronaviridae*, *Picornaviridae*, *Paramyxoviridae*, *Adenoviridae*, and *Parvoviridae*. Bacteria, such as *Streptococcus pneumoniae* (*S*. *pneumoniae*), *Haemophilus influenzae*, *Staphylococcus aureus*, *Moraxella catarrhalis*, *Mycoplasma pneumoniae* (*M*. *pneumoniae*), and *Chlamydia pneumoniae* are the most common involved microbes [3]. However, in general, viral infections are shown to be responsible for about 80% of ARTIs and are the cause of 90% of hospitalizations in children below 5 years of age [4].

According to the localization, ARTIs are divided into 2 categories: lower respiratory tract infections (LRTIs) and upper respiratory tract infections (URTIs). URTIs refer to pathogenic manifestations of rhinosinusitis, tonsillitis, pharyngitis, laryngitis/laryngotracheitis and otitis, while LRTIs include tracheitis, bronchitis, bronchiolitis, and bronchopneumonia [5]. LRTIs are more harmful than URTIs and thus account for most of the serious disease burden. They are the major cause of hospital admissions in young children in the developed world [6].

The current epidemiological situation, patient’s age, clinical symptoms, radiographic and laboratory data and response to treatment may help to differentiate viral from bacterial infections. Even so, no clinical algorithm exists that will distinguish clearly between different causes of childhood respiratory infections [7]. Accurate and rapid diagnostic tests that identify the cause of LRTIs in children can reduce the use of antibiotics, improve the targeted use of drugs and help to control nosocomial transmission [8]. New technologies permit the detection of more than one pathogen in a single probe and are more relevant than non-multiplex approaches such as conventional PCR, viral cell culture and, immunofluorescence or immunochromatography assays. The recently developed commercial diagnostic assays based on multiplex real-time PCR (qPCR/qRT-PCR) allow sensitive and specific detection of a broad panel of conventional and emerging viruses in respiratory tract specimens. Thus, they have been established as a standard method within the past few years. These technological advances have changed the landscape of virus detection and provide the opportunity to improve our understanding of the epidemiology of respiratory viruses [9].

In the Arab Maghreb, few studies have been published describing the detection and the epidemiology of respiratory pathogens [10–12]. In Tunisia for example, studies were limited to few respiratory viruses and focused mostly on mono-detections such as influenza viruses (InfVs) [13, 14], MERS-Coronavirus [15], respiratory syncytial virus (RSV) [16–18] and, metapneumovirus (MPV) [19]. To our knowledge, a single Tunisian study describes the surveillance of InfVs including the detection of a panel of respiratory viruses by multiplex assay, but no information about patient’s clinical profile and its association with viral infection was provided [20].

The purpose of this study was to examine the distribution of viral pathogens, *S*. *pneumoniae* (most common cause for community-acquired bacterial LRTI), and *M*. *pneumoniae* in ARTIs from patients hospitalized between September 2013 and December 2014 in the Sousse area using real-time assays. In addition, we aimed to associate the manifestation of ARTIs with the occurrence of different respiratory pathogens. This study will be helpful for physicians to get a detailed profile about the distribution of respiratory pathogens in Sousse, Tunisia.

## Material and methods

### Ethics statement

Samples enrolled for this study were collected in the context of routine laboratory testing of RSV infection using rapid diagnostic assays (data and results not shown). The study was approved with a formal authorization by the Scientific and Ethical Committee of Farhat Hached University-hospital of Sousse, Tunisia (approval no. IRB 00008931 provided by OHRP).

### Characteristics of study population and sampling

A total of 372 Tunisian cases from September 30, 2013, to December 31, 2014, were enrolled. Patient information (demographic data, medical history, and clinical manifestations) was obtained following a full-filled information form which was made by attending pediatric physicians based on standard clinical criteria. The study includes children aged 1 month to 5 years, hospitalized with URTIs and/or LRTIs, in the pediatric ward (PP) or in intensive care unit of PP ward (PPICU) from Farhat Hached University-hospital of Sousse area. Apparent bronchiolitis was diagnosed in patients with lower respiratory symptoms by wheezing, tachypnea, and signs of respiratory distress such as nasal flaring, intercostal/subcostal retractions, and central cyanosis. Subjects were divided into four age groups: group 1 (G1) includes cases aged 1 to 3 months, group 2 (G2) from 4 to 6 months, group 3 (G3) between 7 and 12 months, and group 4 (G4) from 13 to 60 months with a median patient age of 4.29 months.

Nasopharyngeal aspirations were collected from hospitalized children by a clinical staff from the PP ward and the PPICU. Samples were immediately taken to the laboratory of Microbiology and Immunology at the same hospital where they were diluted in Phosphate-buffered saline (PBS) and centrifuged for 10 min/3584 g/+4°C. The remaining supernatants were divided into aliquots and conserved in -80°C for molecular biology assays.

### Molecular biology assays to identify respiratory pathogens

Molecular biology procedures were accomplished in collaboration with the Division of Virology and the Division of Hygiene and Medical Microbiology, at the Medical University of Innsbruck, Austria.

### Nucleic acids extraction and multiplex qRT-PCR

The QIAsymphony Sp automate (QIAGEN, Cat No./ID: 9001297) and the QIAsymphony DSP virus/Pathogen Mini Kit (QIAGEN GmbH, Austria) were used for the total nucleic acids extraction according to the manufacturer’s instructions. The extraction procedure was carried out from a 600 μl original material resulting in a final eluate of 150 μl. For the detection of respiratory pathogens the FTD Respiratory pathogens 21 Kit (Fast-Track Diagnostics, FTD-2-64) was used which allows the identification of influenza virus A (InfV-A), influenza virus B (InfV-B), influenza virus A (H1N1) swl, rhinovirus (RV), coronavirus NL63, 229E, OC43 and HKU1 (CoV-NL63,CoV-229E, CoV-OC43 and CoV-HKU1), parainfluenza virus 1–4 (PIV-1, PIV-2, PIV-3 and PIV-4), metapneumovirus A and B (MPV A/B), bocavirus (BoV), RSV A/B, adenovirus (AdV), enterovirus (EV), parechovirus (PeV) and *M*. *pneumoniae*. PCR mixture contains: 12.5 μl of PCR buffer, 1.5 μl of Master Mix (primer and probe set 1 to 5), and 1 μl of reverse transcriptase enzyme. All were mixed with 10 μl of nucleic acids for each primer/probe set. Thermocycling was performed on a Rotor-Gene 6000/Q (QIAGEN) using the following program: reverse transcription reaction at 42°C for 15 min, PCR reaction starting by initial denaturation at 94°C for 3 min followed by 40 cycles including a denaturation step at 94°C for 8 sec and annealing and extension step at 60°C for 34 sec.

### *S*. *pneumoniae* qPCR

Pneumococcal genome detection by 16S rDNA qPCR was performed using the Light Cycler Fast Start DNA Mas (#03003248001) kit as per manufacturer’s instructions. The primers and probe used for the *S*. *pneumoniae* identification were designed according to Corless et al [21]. In brief, a total of 20 μl PCR mixture was prepared containing 14 μl Mastermix and 6 μl of extracted sample. The Mastermix contains MgCl_2_, 10x LC-HYBR (a+b), forward primer Strep-F (5’-TGCAGAGCGTCCTTTGGTCTAT-3’), reverse primer Strep-R (5’-CTCTTACTCGTGGTTTCCAACTTGA-3’) (50 μM each), probe Strep-TM (5’-ABI-FAM-TGGCGCCCATAAGCAACACTCGAA-3’-ABI-Tamra) (20 μM), and H_2_O (Roche). Positive and negative controls were included. PCR was carried out on a Light Cycler 2.0 (Roche) with the following program: initial denaturation at 95°C for 7 min, a 50 cycles PCR including a denaturation step at 95°C for 5 sec and an annealing and extension step at 62°C for 45 sec. A final cooling step at 40°C was recommended. PCR results were analyzed using the Light Cycler Software 4.05.

### Statistical analysis

The representation of patient’s data and detected pathogens were analyzed using the SPSS 20.0 statistical package. The relation and differences between pathogens infection rates, clinical manifestations and the statistical calculations were performed using the Chi-square (X^2^) test or the Fisher’s exact test (where cell counts below 5 were encountered in the statistical table) on SPSS. Additionally, to compare total positive, single, and multiple viral infections with patient’s demographic information and the manifestation of ARTIs, the binary logistic regression test was performed when more than 2 categories in the variable were counted. It evaluates the association between the exposure of 2 properties and their outcome (odds ratio: OR) and estimates the associated 95% confidence interval (95% CI) which indicates the degree of uncertainty associated with the OR. A value of p< = 0.05 was considered as significant.

## Results

### Demographic situation and clinical characteristics

During September 2013 and December 2014, 372 children hospitalized with URTIs and/or LRTIs under or equal 5 years old were enrolled. Some patients had an incomplete recovery at the time of discharge, and their data were included as “missing data”. The characteristics of the study population are summarized in Table 1 and S1 Table. Most hospitalized patients were males (64.24%), living in an urban environment (70.69%), and with siblings (70.96%). A total of 72/372 of cases was premature at birth (19.35%) and less than 10.00% were diagnosed with asthma. Almost half of the population was hospitalized between January and March 2014 (49.46%). From total hospitalizations, 12.63% were admitted to the PPICU, the rest were hospitalized in the PP ward (87.36%). The median duration of hospitalization was 8 days in the PP ward, and 1 day at the PPICU. The maximum duration of hospitalization at the PPICU was 13 days and 6.45% of cases were admitted for more than 3 days. The clinical profile of hospitalized subjects showed that the majority of patients were diagnosed for LRTIs (bronchiolitis, 69.35%), while URTIs were characterized by rhinitis (21.23%), laryngitis (4.83%) and pharyngitis (2.68%). Symptoms included mostly dyspnea (75.26%), polypnea (50.80%) and cough (34.94%). Oxygen support was required in 29.03% of hospitalized subjects, 15.59% of patients were diagnosed for respiratory irritation, and 7.79% required mechanical ventilation. A total of 32.25% of patients had a concentration of C-reactive protein (CRP) ranging from equal to higher than 20 milligrams per liter (mg/l). Due to suspicion of bacterial infections, patients were treated with antibiotics (58.87%) and corticosteroids (59.67%). Recovery was defined by response to treatment and when the need for oxygen therapy was considered to be no longer necessary (88.44%). After hospitalization, 8 patients (2.15%) went home without permission, and a total of 23 cases ended fatal (6.18%).

**Table 1:** Demographic characteristics, medical history and clinical manifestations of children hospitalized at the University-hospital of Farhat Hached of Sousse between Sept.2013 and Dec.2014 according to age^a^.

Age groups (months, No. of cases)		G1 (1–3 mo, 159)	G2 (4–6 mo, 67)	G3 (7–12 mo, 88)	G4 (13–60 mo, 58)	Total (372)^b^
**Patient’s characteristics**		**No. (%)**				
**General information**						
**Gender**	**Male**	112 (70.44)	37 (55.22)	60 (68.18)	30 (51.72)	239 (64.24)
	**Female**	47 (29.55)	30 (44.77)	28 (31.81)	28 (48.27)	133 (35.75)
**Origin**	**Rural**	38 (23.89)	20 (29.85)	31 (35.22)	20 (34.48)	109 (29.30)
	**Urban**	121 (76.10)	47 (70.14)	57 (64.77)	38 (65.51)	263 (70.69)
**Sibling**		118 (74.21)	54 (80.59)	57 (64.77)	35 (60.34)	264 (70.96)
**No. of siblings**	**(< = 2)**	87 (54.71)	42 (62.68)	45 (51.13)	25 (43.10)	199 (53.49)
	**(3–7)**	31 (19.49)	12 (19.91)	12 (13.63)	10 (17.24)	65 (17.47)
**Asthma**		19 (11.94)	7 (10.44)	6 (6.81)	3 (5.17)	35 (9.40)
**Prematurity**		26 (16.35)	16 (23.88)	19 (21.59)	11 (18.96)	72 (19.35)
**Seasonal distribution**						
**September-December 2013**		33 (20.75)	18 (26.86)	17 (19.31)	10 (17.24)	78 (20.96)
**January-March 2014**		86 (54.08)	27 (40.29)	45 (51.13)	26 (44.82)	184 (49.46)
**April-June 2014**		22 (13.83)	10 (14.92)	12 (13.63)	6 (10.34)	50 (13.44)
**July-September 2014**		7 (4.40)	5 (7.46)	6 (6.81)	1 (1.72)	19 (5.10)
**October-December 2014**		11 (6.91)	7 (10.44)	8 (9.09)	15 (25.86)	41 (11.02)
**Hospitalization**						
**Duration of hospitalization (days)**	**(1–3)**	33 (20.75)	3 (4.47)	10 (11.36)	8 (13.79)	54 (14.51)
	**(4–7)**	59 (37.10)	31 (46.26)	41 (46.59)	17 (29.31)	148 (39.78)
	**(>7)**	66 (41.50)	33 (49.25)	37 (42.04)	32 (55.17)	168 (45.16)
**Hospitalization in ICU**		23 (14.46)	10 (14.92)	8 (9.09)	6 (10.34)	47 (12.63)
**Duration in ICU (days)**	**(1–3)**	8 (5.03)	5 (7.46)	5 (5.68)	5 (8.62)	23 (6.18)
	**(>3)**	15 (9.43)	5 (7.46)	3 (3.40)	1 (1.72)	24 (6.45)
**Symptoms**						
**Cough**		62 (38.99)	22 (32.83)	26 (29.54)	20 (34.48)	130 (34.94)
**Vomiting**		26 (16.35)	22 (32.83)	16 (18.18)	12 (20.68)	76 (20.43)
**Immuno-deficiency**		8 (5.03)	7 (10.44)	5 (5.68)	7 (12.06)	27 (7.25)
**CRP (mg/l)**	**< 20**	103 (64.77)	33 (49.25)	54 (61.36)	39 (67.24)	229 (61.55)
	**> = 20**	44 (27.67)	30 (44.77)	28 (31.81)	18 (31.03)	120 (32.25)
**URTIs/LRTIs**						
**Rhinitis**		38 (23.89)	19 (28.35)	10 (11.36)	12 (20.68)	79 (21.23)
**Laryngitis**		8 (5.03)	5 (7.46)	1 (1.13)	4 (6.89)	18 (4.83)
**Pharyngitis**		3 (1.88)	4 (5.97)	3 (3.40)	0 (00.00)	10 (2.68)
**Bronchiolitis**		90 (56.60)	46 (68.65)	70 (79.54)	52 (89.65)	258 (69.35)
**Predictive severity symptoms**						
**Respiratory irritation**		21 (13.20)	15 (22.38)	11 (12.50)	11 (18.96)	58 (15.59)
**Apnea**		15 (9.43)	12 (17.91)	10 (11.36)	11 (18.96)	48 (12.90)
**Saturation of O_2_ <94%**		31 (19.49)	17 (25.37)	13 (14.77)	10 (17.24)	71 (19.08)
**Dehydration**	**Absent**	133 (83.64)	52 (77.61)	65 (73.86)	45 (77.58)	295 (79.30)
	**Less severe**	19 (11.94)	8 (11.94)	12 (13.63)	7 (12.06)	46 (12.36)
	**Severe**	7 (4.40)	7 (10.44)	11 (12.50)	6 (10.34)	31 (8.33)
**Post-hospitalization**						
**Recovery**		141 (88.67)	58 (86.56)	82 (93.18)	48 (82.75)	329 (88.44)
**Left against medical advice**		5 (3.14)	1 (1.49)	2 (2.27)	0 (00.00)	8 (2.15)
**Nosocomial infection**		21 (13.20)	13 (19.40)	17 (19.31)	15 (25.86)	66 (17.74)
**Death**		7 (4.40)	5 (7.46)	5 (5.68)	6 (10.34)	23 (6.18)

^a^ The percentages refer to the number of patients in each age group (159 cases in G1 group, 67 infants in G2 group, 88 patients in G3 groups and, 58 children in G4 group).

^b^ The percentages shown in the column “Total” gives the fraction of the total enrolled subjects (No = 372).

### Distribution of respiratory agents

#### Multiplex detection of pathogens

**Fig 1:**
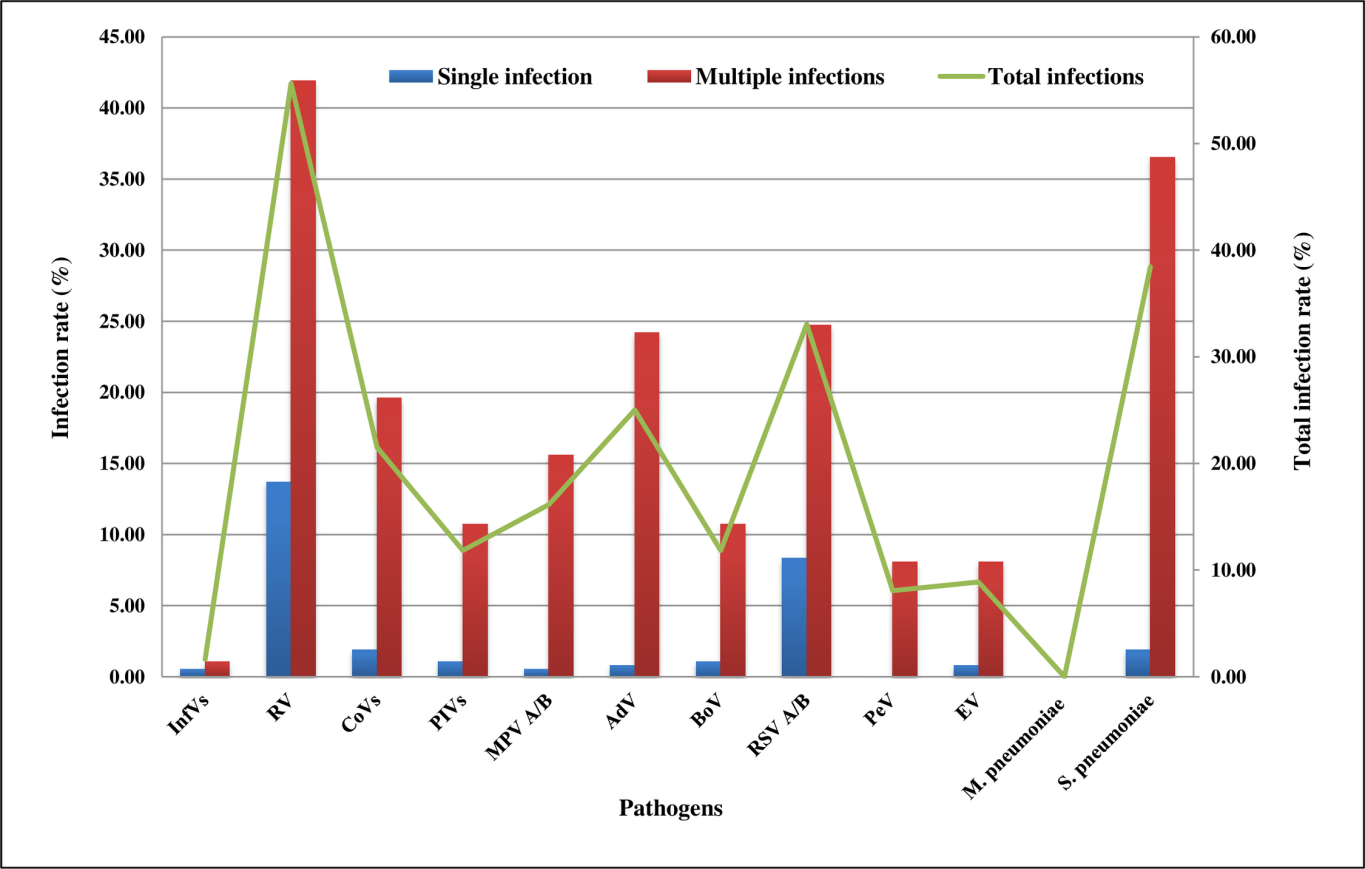
Pathogens etiology within the tested samples. At the left y-axis, the rates of pathogen ARTIs are given. The rates of single infection are shown in blue, and the rates of multiple infections in red. The total infection rates are presented by the green line (right y-axis). InfVs, CoVs, and PIVs types are combined in groups.

A total of 720 pathogens were detected in the 372 tested samples. In 28.76% (107/372) of patients, a single pathogen was found, and in 235 specimens (63.17%) more than 1 pathogen was detected. RV was the most frequently identified pathogen (55.64%, 207/372 samples), followed by RSV-A/B (33.06%, 123/372), AdV (25.00%, 93/372), coronavirus group (CoVs, 21.50%, 80/372), MPV-A/B (16.12%, 60/372), parainfluenza virus group (PIVs) with BoV (11.82%, 44/372 each), EV (8.87%, 33/372), PeV (8.06%, 30/372), and influenza virus group (InfVs, 1.61%, 6/372). Among CoVs and PIVs groups, more than the half were positive for CoV-229E (56.25%, 45/80 total CoVs infections) and PIV-3 (56.81%, 25/44 total PIVs infections), respectively. Not a single *M*. *pneumoniae* infection was found, and in 8.06% of tested samples (30/372), no pathogen was detected by multiplex-qRT-PCR (Fig 1 and Table 2).

**Table 2:** The number of individual viral infection and co-infections detected in hospitalized children in Sousse area, Tunisia (Sept.2013-Dec.2014)^a^.

	InfV-A	InfV-B	InfV-A (H1N1) swl	RV	CoV-NL63	CoV-229E	CoV-OC43	CoV-HKU1	PIV-1	PIV-2	PIV-3	PIV-4	MPV A/B	AdV	BoV	RSV A/B	PeV	EV
**InfV-A**	**2**	0	0	1	0	0	0	1	0	0	0	0	0	1	0	1	0	0
**InfV-B**		**0**	0	0	0	0	0	0	0	0	0	0	0	0	0	0	0	0
**InfV-A (H1N1) swl**			**0**	0	0	0	0	0	0	0	0	0	0	0	0	1	0	0
**RV**				**51**	1	29	1	16	2	1	18	9	**44**	**53**	25	**44**	16	0
**CoV-NL63**					**1**	0	0	0	0	0	0	1	1	1	1	4	2	1
**CoV-229E**						**2**	0	0	0	0	4	1	14	12	5	10	3	3
**CoV-OC43**							**1**	0	0	0	1	0	0	1	1	1	0	0
**CoV-HKU1**								**3**	0	0	4	1	2	8	3	13	5	2
**PIV-1**									**0**	0	0	0	0	0	0	1	1	0
**PIV-2**										**1**	0	0	1	0	0	1	0	0
**PIV-3**											**3**	0	5	4	7	4	2	3
**PIV-4**												**0**	2	2	1	2	3	0
**MPV A/B**													**2**	17	6	8	3	7
**AdV**														**3**	12	27	10	16
**BoV**															**4**	11	3	8
**RSV A/B**																**31**	24	11
**PeV**																	**0**	4
**EV**																		**3**

^a^ The single viral infections are shown in bold, and the most frequent co-infections are underlined in bold.

#### *S*. *pneumoniae* infection

Out of 372 tested samples, 143 (38.44%) were positive for *S*. *pneumoniae* qPCR in which 7 samples were mono-infected by this bacterium (1.88%), and 136 (36.56%) samples had, in addition to *S*. *pneumoniae*, at least one respiratory virus (Fig 1). In more than half of patients with a positive *S*. *pneumoniae* (81/143), the concentrations of CRP remained low (<20 mg/l) indicating that this bacterium is not the causative agent of the observed ARTI. A total of 53 of the *S*. *pneumoniae* positive samples (73.06%) had CRP concentrations equal or above 20 mg/l. Forty-seven of these 53 cases were additionally positive for at least 1 respiratory virus covered by the multiplex qRT-PCR. In 6 further cases with high CRP concentrations (> = 20 mg/l), this bacterium was the single detected pathogen suggesting that these patients were hospitalized due to *S*. *pneumoniae* infection (Table 3).

**Table 3:** Characteristics of *S*. *pneumoniae* infected subjects in relation to viral infection (negative, single and multiple) and CRP concentrations^a^.

*S*. *pneumoniae* infection/CRP estimation	Viral infection			
**CRP concentration (mg/l)**	**Negative**	**Single**	**Multiple**	**Total**
**CRP > = 20**	6	17	30	53
**CRP <20**	1	34	46	81
**Unknown CRP^b^**	0	2	7	9
**Total**	**7**	**53**	**83**	**143**

^a^ From the *S*. *pneumoniae* positive cases, a total of 7 patients are negative for viral infection. Six of these 7 cases had CRP concentrations from equal to higher than 20 mg/l. In addition, 47/143 *S*. *pneumoniae* positive patients had CRP concentrations from equal to higher than 20 mg/l, with single or multiple viral infections.

^b^ Referred to children with an incomplete CRP estimation at the time of discharge.

### Characteristics of infected children

#### Overview of infected children

The demographic and clinical information of infected subjects are described in S2 Table. Infected cases were predominantly males, living in the city (urban environment), staying at home and not in daycare units or in the nursery, and living with siblings. Bronchiolitis was the most characterized LRTI within infected subjects. URTIs were defined by laryngitis and pharyngitis. Symptoms including polypnea, dyspnea, anemia, and dehydration were reported. Frequently, respiratory irritation was found, and oxygen support was required in children with positive viral infection. Previous medical history like asthma, passive smoking, and prematurity at birth are outlined.

#### Age distribution of viral infections

**Fig 2:**
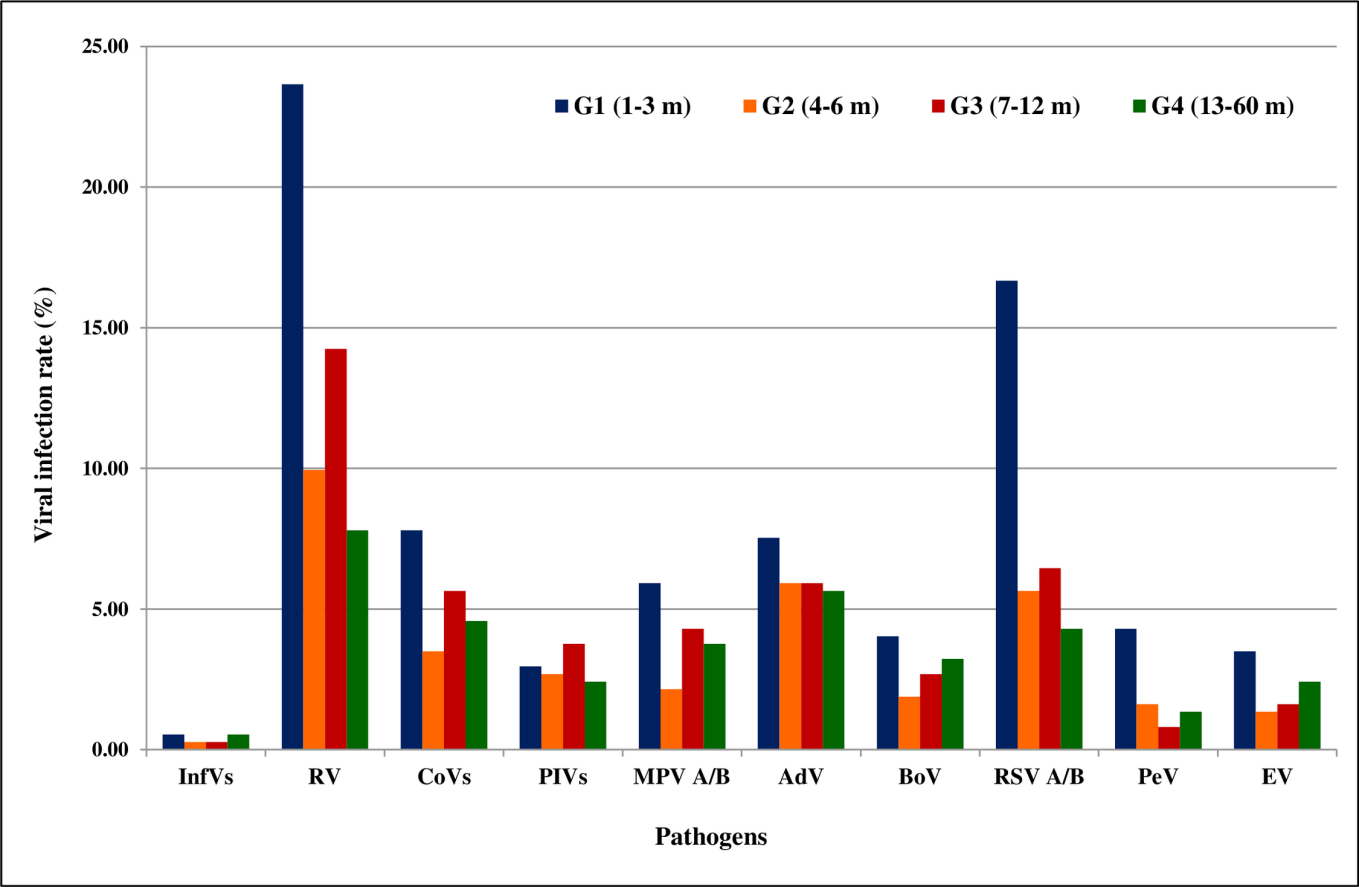
Distribution of viral infection rates in relation to the age of patients. Four age groups are defined: G1 (1–3 m, blue), G2 (4–6 m, orange), G3 (7–12 m, red), and G4 (13–60 m, green). The rates of viral infection are represented in the y-axis, and the x-axis is described by tested viruses.

Fig 2 summarizes the distribution of viral infection rates within the four age groups. Viral infection was more frequent in G1group (76.88% of the 372 tested samples). RV, RSVA/B, CoVs group and MPVA/B were predominant within patients from G1 and G3 groups, while BoV, InfVs group, and EV were most frequently detected in patients belonging to G1 and G4 age groups. PIVs infection was most commonly described in patients aged from 7 to 12 months (G3 group). AdV infection was age-dependent (p<0.05), and was more frequent in children below 2 years old (results not shown). PeV was more frequent in children less than 4 months of age.

#### Association between viral infection and patient’s demographic and clinical characteristics

Tables 4 and 5 summarize the most important patient’s demographic and clinical information found in association with viral infection (total positive, single and multiple). Viral infection increases in subjects living in a humid environment (p = 0.041), and with a primary care in the nursery (p = 0.040). Children breastfeed after birth were at high risk of being infected by a single virus than being infected by multiple viruses (p = 0.001). Multiple infections were found to increase the risk for developing respiratory irritation (p = 0.001).

**Table 4:** Summary of the statistical comparisons between positive viral infection and patient’s medical history, demographic data and clinical manifestations.

Patient’s data		Negative (No = 30) No. (%)	Positive (No = 342) No. (%)	P-value	OR [95% CI]
**Age groups (months)**	G1 (1–3)*	14 (8.80)	145 (91.19)	0.354	1
	**G2 (4–6)**	8 (11.94)	59 (88.05)		0.370 [0.081–1.680]
	**G3 (7–12)**	6 (6.81)	82 (93.18)		0.263 [0.054–1.294]
	**G4 (13–60)**	2 (3.44)	56 (96.55)		0.488 [0.095–2.506]
**Humidity**	No*	19 (6.81)	260 (93.19)	**0.041**	1
	**Yes**	9 (14.75)	52 (85.24)		0.422 [0.181–0.985]
**No. of siblings**	(< = 2)*	19 (9.54)	180 (90.45)	0.400	1
	**(3–7)**	4 (6.15)	61 (93.84)		1.610 [0.527–4.917]
**Primary care in the nursery**	No*	20 (10.92)	163 (89.07)	**0.040**	1
	**Yes**	6 (4.51)	127 (95.49)		2.597 [1.013–6.658]
**Breastfeeding**	No*	24 (7.50)	296 (92.50)	0.406	1
	**Yes**	6 (11.54)	46 (88.46)		0.622 [0.241–1.603]
**Vaccination against Influenza**	No*	28 (8.07)	319 (91.93)	1	1
	**Yes**	1 (4.38)	22 (95.65)		1.931 [0.251–14.864]
**Respiratory irritation**	No*	24 (8.08)	273 (91.92)	1	1
	**Yes**	4 (6.90)	54 (93.10)		1.187 [0.396–3.558]
**Oxygenation duration (days)^a^**	(1–3)*	0 (0.00)	31 (100.00)	N/A	N/A
	**(4–7)**	11 (15.27)	61 (84.72)		N/A
	**(>7)**	0 (0.00)	4 (100.00)		N/A

The statistical calculations comparing total positive viral infection and patient’s demographic information and clinical manifestations were evaluated using SPSS statistical package. The percentages were estimated as a fraction of total cases belonging to each category. P-values were estimated using the X^2^ test or the Fisher’s exact test. For the variables with more than 2 categories (age groups and the duration of oxygenation in days), the binary logistic regression test was performed to determine the reference group, the OR, and the 95% CI. P-values < = 0.05 are represented in bold.

* Represents the reference group used for the statistical associations (e.g. for the variable age groups, G1 was the reference group, and P-value was established comparing the total positive amount of viral infection with G1 vs G2 + G1 vs G3 + G1 vs G4).

^a^ For the variable “oxygenation duration”, the statistical test was not applicable (N/A).

**Table 5:** Summary of the statistical associations comparing single and multiple viral infections with patient’s data.

Patient’s data		Single (No = 107) No. (%)	Multiple (No = 235) No. (%)	P-value	OR [95% CI]
**Age groups (months)**	G1 (1–3)*	55 (37.93)	90 (62.07)	0.125	1
	**G2 (4–6)**	15 (25.42)	44 (74.57)		0.495 [0.244–1.001]
	**G3 (7–12)**	24 (29.26)	58 (70.73)		0.887 [0.378–2.082]
	**G4 (13–60)**	13 (23.21)	43 (76.78)		0.718 [0.328–1.570]
**Humidity**	No*	81 (31.15)	179 (68.84)	0.304	1
	**Yes**	20 (38.46)	32 (61.54)		0.724 [0.391–1.342]
**No. of siblings**	**(< = 2)**	58 (32.22)	122 (67.77)	0.310	1
	**(3–7)**	24 (39.34)	37 (60.65)		0.733 [0.402–1.337]
**Primary care in the nursery**	No*	50 (30.67)	113 (69.32)	0.881	1
	**Yes**	40 (31.50)	87 (68.50)		0.962 [0.583–1.588]
**Breastfeeding**	No*	83 (28.04)	213 (71.96)	**0.001**	1
	**Yes**	24 (52.17)	22 (47.8)		0.357 [0.190–0.672]
**Vaccination against Influenza**	No*	104 (32.60)	215 (67.40)	0.064	1
	**Yes**	3 (13.64)	19 (86.36)		3.064 [0.887–10.586]
**Respiratory irritation**	No*	97 (35.53)	176 (64.47)	**0.001**	1
	**Yes**	7 (13.00)	47 (87.00)		3.700 [1.611–8.502]
**Oxygenation duration (days)^a^**	(1–3)*	6 (19.35)	25 (80.65)	N/A	N/A
	**(4–7)**	20 (32.79)	41 (67.21)		N/A
	**(>7)**	2 (50.00)	2 (50.00)		N/A

The statistical associations between single/multiple viral infections and patient’s demographic information and clinical manifestations were calculated using SPSS statistical package. The percentages were estimated as a fraction of total cases belonging to each category. P-values were estimated using the X^2^ test or the Fisher’s exact test. For the variables with more than 2 categories (age groups and the duration of oxygenation in days), the binary logistic regression test was performed to determine the reference group, the OR and the 95% CI. P-values < = 0.05 are represented in bold.

* Represents the reference group used for the statistical associations (e.g. for the variable age groups, G1 was the reference group, and P-value was established comparing the total positive amount of viral infection with G1 vs G2 + G1 vs G3 + G1 vs G4).

^a^ For the variable “oxygenation duration”, the statistical test was not applicable (N/A).

The statistical comparisons between individual viral infection and patient’s information are summarized in Table 6. RSV A/B and PeV were the most associated pathogens with patient’s demographic situation and the manifestations of ARTIs: RSV A/B infection increases with the increasing number of siblings and occurs more in children with a primary care in the nursery (p<0.05). In addition, RSV A/B was found to increase the need for oxygen support (p = 0.043), the duration of hospitalization in ICU (p = 0.017), and the risk of nosocomial infection (p = 0.031). However, PeV was statistically associated with asthma and anemia (p<0.05). Interestingly, PeV and PIVs group were the single respiratory agents found to increase the risk of death (p = 0.032 and p = 0.013 respectively). RV was found to be statistically associated with a prolonged duration of oxygenation need after hospitalization (p = 0.042), BoV increases the risk for developing pharyngitis (p = 0.003), and EV was statistically related to nosocomial infections (p = 0.049). Infection due to CoVs group was found to increase the risk of bronchiolitis (p = 0.009) and laryngitis (p = 0.017). Vomiting was reported in 20.00% of patients, of which 1/3 were infected by AdV. In all of these cases, co-infections with at least 1 additional virus were found. In addition, AdV was found to be statistically associated with gastroenteritis (p = 0.004). MPV A/B was the single virus not found to be associated with patient’s demographic characteristics and the manifestation of ARTIs. As mentioned above, only 6/372 cases were found to be infected by InfVs. Thus, no statistical comparisons of InfVs and the manifestation of ARTIs were taken into consideration.

**Table 6:** Impact of individual viral infection on patient’s clinical data and demographic information^a^.

Patient’s data/ Pathogens		RV	CoVs	PIVs	MPVA/B	AdV	BoV	RSVA/B	PeV	EV
**Medical history**	**Sibling no (< = 2, >2)**	0.758	0.348	0.469	0.714	0.448	0.283	**0.008**	**0.012**	0.580
	**Primary care in nursery (Yes, No)**	0.197	0.214	0.307	0.282	0.211	0.235	**0.010**	0.981	0.356
	**Primary care in daycare (Yes, No)**	0.509	0.604	0.100	0.081	0.321	0.324	0.751	0.239	0.365
	**Household (Yes, No)**	0.274	0.763	0.263	0.215	0.182	0.535	0.367	0.198	0.362
	**Breastfeeding (Yes, No)**	0.560	0.427	**0.017**	0.169	0.300	0.319	0.127	0.783	0.435
	**Artificial feeding (Yes, No)**	0.446	0.055	1.000	0.356	0.517	0.490	**0.004**	0.234	1.000
	**Mixed feeding (Yes, No)**	0.605	0.291	0.270	0.588	0.122	0.458	0.553	**0.012**	0.098
	**Asthma (Yes, No)**	0.230	0.110	0.052	0.408	0.926	0.286	0.866	**0.004**	0.755
	**Passive smoking (Yes, No)**	0.804	0.262	0.924	0.418	0.792	0.814	0.232	0.428	0.564
	**Prematurity (Yes, No)**	0.438	0.869	0.068	0.890	0.762	0.153	0.822	0.926	0.522
**Hospitalization**	**Hospitalization in ICU (Yes, No)**	0.790	0.423	0.238	0.502	0.528	0.238	0.628	0.246	0.406
	**Duration of hospitalization in ICU (1–3 d, >3 d)**	0.454	0.674	0.266	0.176	0.703	0.590	**0.017**	1.000	1.000
	**Oxygen therapy (Yes, No)**	0.157	0.770	0.461	0.322	0.326	0.703	**0.043**	0.054	0.915
	**Duration of oxygen therapy (1–3 d, 4–7 d, >7 d)**	**0.042**	0.535	0.088	0.102	0.714	0.893	0.296	0.863	0.075
**Side symptoms**	**Anemia (Yes, No)**	0.222	0.751	0.825	0.578	0.558	0.382	0.293	**0.003**	0.175
	**Vomiting (Yes, No)**	0.073	0.898	**0.049**	0.552	0.205	0.710	0.434	0.728	0.552
	**Gastroenteritis (Yes, No)**	0.697	0.613	0.533	1.000	**0.004**	1.000	0.668	1.000	1.000
**Predictive severity factors**	**Respiratory irritation (Yes, No)**	0.112	0.539	0.613	0.608	0.539	0.221	0.209	0.290	0.294
	**Apnea (Yes, No)**	0.258	0.375	0.561	0.841	0.435	0.755	0.233	0.781	0.782
	**Saturation of O**_**2**_ **<94% (Yes, No)**	0.697	0.680	0.843	0.351	0.059	0.545	**0.016**	0.097	0.929
	**Dehydration (Yes, No)**	0.737	0.377	0.512	0.749	0.494	0.411	0.582	1.000	0.387
**Post-hospitalization**	**Nosocomial infection (Yes, No)**	0.505	0.411	**0.000**	0.895	0.113	0.846	**0.031**	0.564	**0.049**
	**Death (Yes, No)**	0.403	0.998	**0.013**	0.559	0.907	0.739	0.542	**0.032**	0.708
**Upper/Lower ARTIs**	**Bronchiolitis (Yes, No)**	0.437	**0.009**	0.118	0.300	0.897	0.598	0.429	0.739	0.124
	**Pharyngitis (Yes, No)**	0.195	0.127	1.000	0.667	0.716	**0.003**	0.736	0.573	0.610
	**Laryngitis (Yes, No)**	0.142	**0.017**	0.458	0.328	0.408	1.000	0.293	0.648	1.000
	**Rhinitis (Yes, No)**	0.301	0.141	0.696	0.567	0.915	0.909	0.462	0.109	0.367

^a^ The reference group used for the statistical calculations constituted of the total amount of viral infections which was associated with patient’s clinical manifestations and demographic data. P-values were calculated using the X^2^ test or the Fisher’s exact test on SPSS considering a value of p< = 0.05 as significant (underlined in bold). InfVs was not included in the statistical comparisons due to the small number of infected cases (6 InfVs infections). Subjects with “missing data” were not included in the statistical calculations.

## Discussion

This study analyzed the frequencies of ARTIs by respiratory viruses (18 pathogens) and 2 common bacteria (*M*. *pneumoniae* and *S*. *pneumoniae*) using sensitive molecular detection techniques. Collected specimens from 372 hospitalized infants and children aged between 1 month and 5 years from Sousse area in Tunisia were enrolled. In 342 out of 372 tested samples, respiratory pathogens were detected. This high rate of infection (91.93%) is in line with previous studies [22, 23], although others findings reported lower infection rates [24, 25]. The differences in detection rates may depend on a variety of factors including heterogeneity of study patients, differences in clinical manifestations diagnosed in patients, the number of tested respiratory pathogens and especially the tests used for their detection [26].

The most common viruses detected in this study were RV and RSV A/B responsible for 55.64% and 33.06% of infections, respectively. Both of these pathogens were also identified by other studies as the major etiologic agents for ARTIs in infants and young children [27, 28]. RSV is well established as a common agent of ARTIs, especially in the manifestation of bronchiolitis. In addition, studies from Algeria, Morocco, and Tunisia reported RSV infection rates between 18.00% and 29.80% [10–12, 18]. In contrast to RSV, few studies were published describing RV epidemiology in the Arab Maghreb. Similar to our findings, Jroundi and collaborators detected a high rate of RV infection (53.00%) in Moroccan children hospitalized with ARTIs [11]. In another hand, a recent study describing the circulation of respiratory viruses in Tunisia over 3 consecutive years found that RV infection was predominant in only 1 of the 3 years, while RSV was predominant in the other 2 years. Such findings indicate a possible fluctuation of RV and RSV circulation [20]. The interpretation of the presence and the pathogenic role of RV in LRTIs remain unclear. According to Vandini et al, RV is the second most common cause of viral bronchiolitis and the most common virus associated with wheezing in children aged 1 to 2 years [29]. However, the detection of RV in asymptomatic children, or its long persistence after an acute respiratory infection has been also described [30].

The AdV is recognized as a compelling cause of gastrointestinal, ophthalmologic, genitourinary and neurological syndromes. It is also known as an important agent of upper and lower respiratory tract infections in children. In the present study, AdV was found in 25.00% of the tested samples. Besides its role in the induction of ARTIs, AdV was statistically associated with gastroenteritis manifestation (viral tropism, p = 0.004). These findings are in accordance with other studies describing AdV acute infections requiring hospitalization [31, 32]. Similar to AdV, CoVs group including CoV-229E, CoV-HKU1, CoV-NL63, and CoV-OC43, was detected in 21.50% of the samples, which is in contrast to other publications reporting that CoVs are rarely detected in ARTIs [33, 34].

Other pathogens such as MPV A/B, PIVs group (including PIV-1 to PIV-4), BoV, EV, PeV, and InfVs group (InfV-A, InfV-B, and InfV-A (H1N1) swl), had detection rates ranging from 16.12% to 1.61%. The rare presence of Influenza infection in this study (1.61% infection rate) may support a published meta-analysis indicating that very young children (<2 years of age) are at significantly lower risk for hospital admission caused by influenza infection compared with the other age groups (84.40% of our population was aged under 12 months) [35].

In contrary to published Tunisian studies, which primarily focused on detection of a single respiratory agent in ARTIs in children [15, 18], this study covers hospitalized, and not-preselected children. We aimed to identify a causative agent of ARTIs and to link these pathogens with the severity of symptoms and available demographic data. A high percentage of cases suffering severe symptoms like apnea (12.90%), respiratory irritation (15.59%), polypnea (50.80%), oxygen saturation <94% (19.08%) and severe dehydration (8.33%) were reported. Complications such as vomiting were found in 20.43% of patients. Furthermore, the present study detected viral co-infections in 235 out of 372 tested samples. RV and RSV A/B were the most common pathogens found in combination with other viruses (RV-AdV in 53/235 total co-infections, then RV-RSV A/B and RV-MPV A/B in 44/235 co-infected samples, each). This finding is supported by Cebey-López et al, who studied viral co-infections in a pediatric environment in which RSV and RV are the most detected pathogens [36].

Although the size of the samples is limited, the present study allows us to analyze putative associations between multiple infections and clinical manifestations (Table 5) as the probability of being infected by the combination of at least 2 respiratory viruses increases when children were breastfed after birth, and the risk for developing respiratory irritation raised with viral multiple infections. The association of multiple infections with increased severity of the course of infection is controversial in the literature. Some reports showed no association between multiple virus infections and disease severity, whereas the findings of others indicated that clinical manifestations and illness severity were more prevalent in multiple infected patients, especially in co-infections with RSV [37–39].

In the same way, a statistical association was observed when comparing individual viral infection and patient’s clinical manifestations (Table 6). RSV was the most associated virus with respiratory illness reflected by the need for oxygen support, the duration of hospitalization in ICU and nosocomial infections among hospitalized children (p<0.05). PeV came after and was statistically associated with asthma, anemia, and death (p<0.05). Human enterovirus was reported in 8.87% of cases, supporting studies from Vietnam [40], Europe [41] and Southern Asia [42], in which EV was detected in a large fraction of children with ARTIs. Similar to PIVs group and RSV A/B, EV was found to increase the risk of nosocomial infection in hospitalized children (p<0.05).

Beside virus-specific nucleic acids, a total of 143/372 ARTIs were found to be positive for *S*. *pneumoniae*. In most of *S*. *pneumoniae* positive patients (81/143), CRP concentrations were low (<20 mg/l) indicating that the detected bacterial DNA is not representing the causative agent for the observed ARTIs, but may demonstrate a colonization status. *S*. *pneumoniae*-carriage rates in children of 60.00% up to 93.00% in low- and lower-middle-income countries have been reported [43, 44]. In our study 47 cases from a total of 53 *S*. *pneumoniae* positive PCR and a concentration of CRP equal or higher than 20 mg/l; viral RNA/DNA was additionally detected, indicating a possible bacterial co-infection. On the other hand, increased colonization rates of *S*. *pneumoniae* in children during RSV epidemics have been reported [45]. In only 6/53 children with CRP concentration values equal or higher than 20mg/l and *S*. *pneumoniae* detection, no virus was found in our study, leading to the interpretation that these children were hospitalized due to *S*. *pneumoniae*-associated ARTIs. Pneumococcal vaccination is available in many countries. However, in Tunisia, there is no national vaccination program against pneumococcal in public hospitals. Vaccination is available only in the private hospitals and thus is not accessible to all population [46].

## Conclusion

Acute viral respiratory tract infections continue to be a worldwide health concern. The presentation of clinical symptoms does not often enable clinicians to discriminate between viral or bacterial pathogens. The recommended early and easy use of antibiotics is not effective in viral ARTIs, and can only prevent the occurrence of bacterial super-infection. In this context, viral diagnosis can prevent the use of unnecessary costly antibiotics. For this reason, rapid and reliable diagnostic tools are required for intervention with the appropriate infection treatment measures. This study aimed to profile a large number of viruses and the most common bacteria in the respiratory tract of infected children in Sousse area in Tunisia using the multiplex qRT-PCR for virus’s genome detection and the qPCR assays for bacteria identification. The results of the study should provide a better understanding of the viral/bacterial etiology in this region.

## Supporting information

S1 Table Characteristics of hospitalized children at Farhat Hached University-hospital of Sousse, Tunisia, between 2013 and 2014 by age groups^a^. ^a^ The table summarizes patient’s data according to age distribution. The percentages were calculated as the fraction of total cases from each age group (159 cases in G1 group, 67 infants in G2 group, 88 patients in G3 group, and 58 children in G4 group). The rates described in the column “Total” were calculated dividing on the number of total cases (No = 372). ^b^ Antibiotics. ^c^ Hydrocortisone (HSHC: hydrocortisone hemisuccinate).
(PDF)

S2 Table Distribution of demographic/environmental data, medical history and clinical manifestations between infected patients according to individual viral infection. ^a^ InfV-A, InfV-B, and InfV-A (H1N1) swl were grouped in InfVs group. ^b^ CoV- NL63, CoV-229E, CoV-OC43, and CoV-HKU1 were included in CoVs group. ^c^ Para influenza virus 1–4 were combined in PIVs group. ^d^ The percentages were calculated as the fraction of total infected cases in each column (e.g. dividing on 6 InfVs infected cases).
(PDF)
