# Supplementary material for: Epidemiology and clinical profile of pathogens responsible for the hospitalization of children in Sousse area, Tunisia
Source: PLoS One. 2017 Nov 17;12(11):e0188325. doi: 10.1371/journal.pone.0188325 (PMC5693464; doi:10.1371/journal.pone.0188325)
Supplement: S1 Table — a The table summarizes patient’s data according to age distribution. The percentages were calculated as the fraction of total cases from each age group (159 cases in G1 group, 67 infants in G2 group, 88 patients in G3 group, and 58 children in G4 group). The rates described in the column “Total” were calculated dividing on the number of total cases (No = 372). b Antibiotics. c Hydrocortisone (HSHC: hydrocortisone hemisuccinate). (PDF) [file pone.0188325.s001.pdf]

S1 Table. Characteristics of hospitalized children at Farhat Hached University-hospital of Sousse, Tunisia, between 2013 and 2014 by age groups[a].

Age (age group, cases no)		G1 (1-3 mo, 159)	G2 (4-6 mo, 67)	G3 (7-12 mo, 88)	G4 (13-60 mo, 58)	Total (372)
**Patient's characteristics**		No (%)				
**Clinical manifestations**						
**Polypnea**		82 (51.57)	36 (53.73)	38 (43.18)	33 (56.89)	189 (50.80)
**Dyspnea**		129 (81.13)	51 (76.11)	60 (68.18)	40 (68.96)	280 (75.26)
**Respiratory co-infections**						
**Gastroenteritis**		2 (1.25)	2 (2.98)	2 (2.27)	0 (00.00)	6 (1.61)
**Angina**		1 (0.62)	0 (00.00)	1 (1.13)	0 (00.00)	2 (0.53)
**Clinical support**						
**Oxygen therapy**		53 (33.33)	21 (31.34)	20 (22.72)	14 (24.13)	108 (29.03)
**Oxygen therapy**	**Mask**	5 (3.14)	1 (1.49)	1 (1.13)	2 (3.44)	9 (2.41)
	**Glasses**	47 (29.55)	20 (29.85)	18 (20.45)	12 (20.68)	97 (26.07)
**Oxygenation duration (No. of days)**	**(1-3)**	15 (9.43)	4 (5.97)	6 (6.81)	3 (5.17)	28 (7.52)
	**(4-7)**	36 (22.64)	17 (25.37)	11 (12.50)	11 (18.96)	75 (20.16)
	**(> 7)**	2 (1.25)	0 (00.00)	1 (1.13)	0 (00.00)	3 (0.80)
**Ventilation**		5 (3.14)	7 (10.44)	10 (11.36)	7 (12.06)	29 (7.79)
**Treatment**						
**ATB[b]**		84 (52.83)	45 (67.16)	55 (62.50)	35 (60.34)	219 (58.87)
**Corticosteroids**		97 (61.00)	40 (59.70)	52 (59.09)	33 (56.89)	222 (59.67)
**Corticosteroids duration (days)**	**(1-3)**	29 (18.23)	10 (14.92)	8 (9.09)	6 (10.34)	53 (14.24)
	**(4-7)**	68 (42.76)	31 (46.26)	45 (51.13)	28 (48.27)	172 (46.23)
**HSHC[c]**		69 (43.39)	35 (52.23)	44 (50.00)	26 (44.82)	174 (46.77)
**Solumedrol**		20 (12.57)	5 (7.46)	8 (9.09)	5 (8.62)	38 (10.21)
**Pulmicort**		18 (11.32)	12 (17.91)	16 (18.18)	12 (20.68)	58 (15.59)
**Solupred**		11 (6.91)	1 (1.49)	5 (5.68)	3 (5.17)	20 (5.37)
**Alergosone**		4 (2.51)	1 (1.49)	2 (2.27)	0 (00.00)	7 (1.88)

[a] The table summarizes patient's data according to age distribution. The percentages were calculated as the fraction of total cases from each age group (159 cases in G1 group, 67 infants in G2 group, 88 patients in G3 group, and 58 children in G4 group). The rates described in the column "Total" were calculated dividing on the number of total cases (No=372).

[b] Antibiotics.

[c] Hydrocortisone (HSHC: hydrocortisone hemisuccinate).
