# Supplementary material for: Epidemiology and clinical profile of pathogens responsible for the hospitalization of children in Sousse area, Tunisia
Source: PLoS One. 2017 Nov 17;12(11):e0188325. doi: 10.1371/journal.pone.0188325 (PMC5693464; doi:10.1371/journal.pone.0188325)
Supplement: S2 Table — a InfV-A, InfV-B, and InfV-A (H1N1) swl were grouped in InfVs group. b CoV- NL63, CoV-229E, CoV-OC43, and CoV-HKU1 were included in CoVs group. c Para influenza virus 1–4 were combined in PIVs group. d The percentages were calculated as the fraction of total infected cases in each column (e.g. dividing on 6 InfVs infected cases). (PDF) [file pone.0188325.s002.pdf]

S2 Table. Distribution of demographic/environmental data, medical history and clinical manifestations of infected patients according to individual viral infection.

Pathogens		InfVs[a]	RV	MPV A/B	AdV	BoV	RSV A/B	PeV	EV	CoVs[b]	PIVs[c]
**Infected patients**		**6**	**207**	**60**	**93**	**44**	**123**	**30**	**33**	**80**	**44**
**Total samples (No=372)**		**No (%)[d]**									
**Demography/environment**											
**Sex**	**Male**	5 (83.33)	137 (66.18)	40 (66.66)	59 (63.44)	29 (65.90)	81 (65.85)	19 (63.33)	21 (63.63)	53 (66.25)	31 (70.45)
	**Female**	1 (16.66)	70 (33.81)	20 (33.33)	34 (36.55)	15 (34.09)	42 (34.14)	11 (36.66)	12 (36.36)	27 (33.75)	13 (29.54)
**Origin**	**Rural**	2 (33.33)	61 (29.46)	20 (33.33)	32 (34.40)	12 (27.27)	35 (28.45)	9 (30.00)	5 (15.15)	24 (30.00)	12 (27.27)
	**Urban**	4 (66.66)	146 (70.53)	40 (66.66)	61 (65.59)	32 (72.72)	88 (71.54)	21 (70.00)	28 (84.84)	56 (70.00)	32 (72.72)
**Sibling**		3 (50.00)	150 (72.46)	36 (60.00)	59 (63.44)	35 (79.54)	84 (68.29)	24 (80.00)	20 (60.60)	51 (63.75)	31 (70.45)
**Sitting situation**	**Nursery**	2 (33.33)	76 (36.71)	23 (38.33)	38 (40.86)	18 (40.90)	34 (27.64)	11 (36.66)	15 (45.45)	34 (42.50)	18 (40.90)
	**Daycare**	2 (33.33)	88 (42.51)	20 (33.33)	35 (37.63)	14 (31.81)	52 (42.27)	10 (33.33)	12 (36.36)	33 (41.25)	13 (29.54)
	**Household**	2 (33.33)	124 (59.90)	36 (60.00)	52 (56.98)	25 (56.81)	69 (56.09)	20 (66.66)	19 (57.57)	49 (61.25)	25 (56.81)
**Feeding**	**Breast-feeding**	0 (00.00)	27 (13.04)	5 (8.33)	10 (10.75)	4 (9.09)	22 (17.88)	3 (10.00)	6 (18.18)	9 (11.25)	1 (2.27)
	**Artificial**	0 (00.00)	10 (4.83)	5 (8.33)	4 (4.30)	1 (2.27)	13 (10.56)	3 (10.00)	1 (0.03)	1 (1.25)	2 (4.54)
	**Mixed**	1 (16.66)	54 (26.08)	18 (30.00)	31 (33.33)	14 (31.81)	31 (52.20)	14 (46.66)	13 (39.39)	18 (22.50)	15 (34.09)
**Medical history**											
**Prematurity**		1 (16.66)	43 (20.77)	12 (20.00)	19 (20.43)	5 (11.36)	23 (18.69)	6 (20.00)	5 (15.15)	16 (20.00)	13 (29.54)
**Asthma**		1 (16.66)	23 (11.11)	4 (6.66)	9 (9.67)	2 (4.54)	11 (8.94)	8 (26.66)	2 (6.06)	11 (13.75)	8 (18.18)
**Passive smoking**		1 (16.66)	79 (38.16)	20 (33.33)	36 (38.70)	16 (36.36)	50 (40.65)	13 (43.33)	11 (33.33)	25 (31.25)	17 (38.63)
**Vaccination against Influenza**		4 (66.66)	10 (4.83)	4 (6.66)	7 (7.52)	3 (6.81)	4 (3.25)	5 (16.66)	2 (6.06)	5 (6.25)	5 (11.36)
**Hospitalization**											
**ICU hospitalization**		1 (16.66)	27 (13.04)	6 (10.00)	10 (10.75)	8 (18.18)	17 (13.82)	6 (20.00)	2 (6.06)	8 (10.00)	8 (18.18)
**Symptoms**											

**Cough**		3 (50.00)	76 (36.71)	24 (40.00)	34 (36.55)	14 (31.81)	38 (30.89)	8 (26.66)	12 (36.36)	27 (33.75)	15 (34.09)
**Anemia**		1 (16.66)	112 (54.10)	29 (48.33)	49 (52.68)	26 (59.09)	62 (50.40)	22 (73.33)	21 (63.63)	40 (50.00)	24 (54.54)
**Dehydration**		0 (00.00)	44 (21.25)	16 (26.66)	18 (19.35)	14 (31.81)	27 (21.95)	7 (23.33)	5 (15.15)	23 (28.75)	10 (22.72)
**Dehydration**	**Severe**	0 (00.00)	17 (8.21)	7 (11.66)	6 (6.45)	7 (15.90)	12 (9.75)	3 (10.00)	3 (9.09)	11 (13.75)	5 (11.36)
	**Less severe**	0 (00.00)	27 (13.04)	9 (15.00)	12 (12.90)	7 (15.90)	15 (12.19)	4 (13.33)	2 (6.06)	12 (15.00)	5 (11.36)
**Immune deficiency**		1 (16.66)	12 (5.79)	3 (5.00)	10 (10.75)	6 (13.63)	10 (8.13)	3 (10.00)	2 (6.06)	5 (6.25)	4 (9.09)
**Respiratory irritation**		2 (33.33)	38 (18.35)	8 (13.33)	13 (13.97)	10 (22.72)	15 (12.19)	7 (23.33)	3 (9.09)	14 (17.5)	8 (18.18)
**Polypnea**		2 (33.33)	107 (51.69)	33 (55.00)	52 (55.91)	21 (47.72)	68 (55.28)	16 (53.33)	15 (45.45)	47 (58.75)	24 (54.54)
**Dyspnea**		4 (66.66)	162 (78.26)	46 (76.66)	69 (74.19)	31 (70.45)	95 (77.23)	25 (83.33)	24 (72.72)	63 (78.75)	30 (68.18)
**Apnea**		2 (33.33)	23 (11.11)	8 (13.33)	14 (15.05)	5 (11.36)	12 (9.75)	3 (10.00)	3 (9.09)	8 (10.00)	7 (15.90)
**Saturation of O2 <94%**		2 (33.33)	38 (18.35)	9 (15.00)	24 (25.80)	10 (22.72)	32 (26.01)	9 (30.00)	6 (18.18)	14 (17.50)	9 (20.45)
**Ventilation**		0 (00.00)	17 (8.21)	5 (8.33)	8 (8.60)	4 (9.09)	9 (7.31)	4 (13.33)	0 (00.00)	9 (11.25)	6 (13.63)
**Oxygen therapy**		1 (16.66)	55 (26.57)	14 (23.33)	24 (25.80)	12 (27.27)	44 (35.77)	14 (46.66)	10 (30.30)	22 (27.50)	11 (25.00)
**Upper/lower ARTIs**											
**Bronchiolitis**		5 (83.33)	147 (71.01)	45 (75.00)	64 (68.81)	29 (65.90)	82 (66.66)	20 (66.66)	19 (57.57)	65 (81.25)	35 (79.54)
**Rhinitis**		1 (16.66)	40 (19.32)	11 (18.33)	20 (21.50)	10 (22.72)	29 (23.57)	10 (33.33)	9 (27.27)	12 (15.00)	10 (22.72)
**Laryngitis**		0 (00.00)	7 (3.38)	1 (1.66)	6 (6.45)	2 (4.54)	8 (6.50)	2 (6.66)	1 (3.03)	0 (00.00)	3 (6.81)
**Pharyngitis**		0 (00.00)	8 (3.86)	2 (3.33)	3 (3.22)	5 (11.36)	4 (3.25)	1 (3.33)	1 (3.03)	0 (00.00)	1 (2.27)
**Respiratory co-infections**											
**Gastro-enteritis**		0 (00.00)	4 (1.93)	1 (1.66)	5 (5.37)	0 (00.00)	1 (0.81)	0 (00.00)	0 (00.00)	2 (2.50)	1 (2.27)
**Angina**		0 (00.00)	1 (0.48)	0 (00.00)	1 (1.07)	0 (00.00)	1 (0.81)	1 (3.33)	0 (00.00)	1 (1.25)	0 (00.00)
**Bacteriology**											
**Bacteria super-infection**		0 (00.00)	48 (23.18)	14 (23.33)	21 (22.58)	10 (22.72)	31 (25.20)	11 (36.33)	10 (30.30)	12 (15.00)	15 (34.09)
**CRP <20 mg/l**		5 (83.33)	127 (61.35)	38 (63.33)	53 (56.98)	24 (54.54)	77 (62.60)	19 (63.33)	20 (60.60)	53 (66.25)	23 (52.27)
**Post-hospitalization**											
**Nosocomial infection**		0 (00.00)	39 (18.84)	11 (18.33)	11 (11.82)	8 (18.18)	15 (12.19)	7 (23.33)	2 (6.06)	17 (21.25)	17 (38.63)

**Recovery**	6 (100.00)	186 (89.85)	52 (86.66)	83 (89.24)	38 (86.36)	108 (87.80)	27 (90.00)	31 (93.93)	69 (86.25)	38 (86.36)
**Death**	0 (00.00)	11 (5.31)	5 (8.33)	6 (6.45)	3 (6.81)	9 (7.31)	5 (16.66)	1 (3.03)	5 (6.25)	7 (15.90)
**Left against medical advice**	0 (00.00)	4 (1.93)	0 (00.00)	3 (3.22)	1 (2.27)	1 (0.81)	0 (00.00)	0 (00.00)	2 (2.50)	0 (00.00)
**Viral infection**										
**Single**	2 (33.33)	1 (0.48)	2 (3.33)	3 (3.22)	4 (9.09)	31 (25.20)	0 (00.00)	3 (9.09)	7 (8.75)	4 (9.09)
**Multiple**	4 (66.66)	5 (2.41)	58 (96.66)	90 (69.77)	40 (90.90)	92 (74.79)	30 (100.00)	30 (90.90)	73 (91.25)	40 (90.90)

[a] InfV-A, InfV-B and InfV-A (H1N1) swl were grouped in InfVs group.

[b] CoV- NL63, CoV-229E, CoV-OC43 and CoV-HKU1 were included in CoVs group.

[c] Para influenza virus 1-4 were combined in PIVs group.

[d] The percentages were calculated as the fraction of total infected cases in each column (e.g. dividing on total 6 InfVs infected cases).
